# Retraction Note to: Mental health status, and suicidal thoughts and behaviors of migrant children in eastern coastal China in comparison to urban children: a cross-sectional survey

**DOI:** 10.1186/s13034-020-0311-2

**Published:** 2020-01-21

**Authors:** Jingjing Lu, Feng Wang, Pengfei Chai, Dongshuo Wang, Lu Li, Xudong Zhou

**Affiliations:** 1 The Institute of Social and Family Medicine, School of Public Health, Zhejiang University, 866 Yuhangtang Rd., Hangzhou, 310058 Zhejiang People’s Republic of China; 2 Yinzhou District CDC, 1221 Xueshi Rd., Ningbo, 315199 Zhejiang People’s Republic of China; 3 Oxford Road, SG16 Samuel Alexander Building, Manchester, M13 9PL UK

## Retraction to: Child Adolesc Psychiatry Ment Health (2018) 12:13 10.1186/s13034-018-0219-2

The authors have retracted this article [1] because they do not have documentation of approval of their study by the ethics committee.

All authors agree with this retraction.
